# Investigating ultrafast carrier dynamics in perovskite solar cells with an extended π-conjugated polymeric diketopyrrolopyrrole layer for hole transportation†

**DOI:** 10.1039/c9ra10009a

**Published:** 2020-02-12

**Authors:** Chandramouli Kulshreshtha, Arul Clement, Torbjörn Pascher, Villy Sundström, Piotr Matyba

**Affiliations:** Department of Physics, Umeå University Umeå 90187 Sweden chandramouli.kulshreshtha@umu.se piotr.matyba@umu.se; Swanson School of Engineering, University of Pittsburgh 3700 O'Hara Street Pittsburgh PA 15261 USA; Department of Chemical Physics, Lund University Lund 22362 Sweden

## Abstract

Here, we show a new diketopyrrole based polymeric hole-transport material (PBDTP-DTDPP, (poly[[2,5-bis(2-hexyldecyl)-2,3,5,6-tetrahydro-3,6-dioxopyrrolo[3,4-*c*]pyrrole-1,4-diyl]-*alt*-[[2,2′-(4,8-bis(4-ethylhexyl-1-phenyl)-benzo[1,2-*b*:4,5-*b*′]dithiophene)bis-thieno[3,2-*b*]thiophen]-5,5′-diyl]])) for application in perovskite solar cells. The material performance was tested in a solar cell with an optimized configuration, FTO/SnO_2_/perovskite/PBDTP-DTDPP/Au, and the device showed a power conversion efficiency of 14.78%. The device charge carrier dynamics were investigated using transient absorption spectroscopy. The charge separation and recombination kinetics were determined in a device with PBDTP-DTDPP and the obtained results were compared to a reference device. We find that PBDTP-DTDPP enables similar charge separation time (<∼4.8 ps) to the spiro-OMeTAD but the amount of nongeminate recombination is different. Specifically, we find that the polymeric PBDTP-DTDPP hole-transport layer (HTL) slows-down the second-order recombination much less than spiro-OMeTAD. This effect is of particular importance in studying the charge transportation in optimized solar cell devices with diketopyrrole based HTL materials.

## Abstract 2

Diketopyrrole based hole-transport organic semiconductor was employed in perovskite solar cells and charge carrier dynamics was explained.

## Graphical abstract

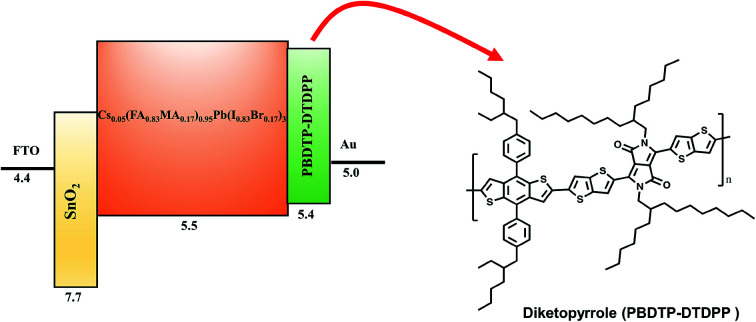

## Introduction

Single-junction hybrid metal–organic perovskite solar cells (PSC) have recently reached ∼22.7% power conversion efficiency (PCE).^1–3^ The impressive performance was enabled by the use of perovskite materials as the absorbers and charge transporters in the photovoltaic device.^4^ These solar cells took significant efforts towards optimizing the device architecture with proper stoichiometry and choice of proper materials for hole or electron transport. Device designs with SnO_2_ or TiO_2_ as the ETM have worked well^5,6^ however there is an increasing demand for replacing, spiro-OMeTAD, as hole transport material (HTM) with cheaper and more stable materials. The problem with spiro-OMeTAD is that it has a tendency to crystallize at 85 °C and needs dopants to enhance the material conductivity^7,8^ which in turn, leads to erosion of the perovskite layer. Several hole-transport materials have been synthesized and investigated, and a wide number include small molecule, polymer, metal–organic compounds *etc.* Sally *et al.* investigated two new HTMs based on dithieno[3,2-*b*:2′,3′-*d*]pyrrole (DTP) derivatives and demonstrated ∼18.2% PCE.^9^ Another very common HTM, PEDOT:PSS was treated with urea which controls phase separation between PEDOT and PSS segments with better morphology and thus gives 18.8% PCE.^10^ On a similar note, ITO free PSCs were fabricated using plasma treated PEDOT:PSS with an efficiency of 10.5%.^11^ The removal of PSS in PEDOT:PSS provides a PCE of about 18.18% due to increase in perovskite crystal grain size.^12^ Conjugated polymeric HTMs have proven to be good alternatives to spiro-OMeTAD due to their better hole-collection ability.^13^ The P3HT polymer as an interlayer achieves 6.49% with better hole-extraction capability in CsPbBr_3_ solar cell devices.^14^ Another polymer interlayer, PBDTT-FTTE achieved 11.6% PCE whereas PDPP3T polymer shows 12.32% PCE with slower degradation and better device stability.^15,16^

Therefore, the properties of different polymer based HTMs and the working mechanism including the hole-injection, charge separation and accumulation, light-and-field induced ion movement^17–19^ with perovskite layer need to be better understood. The interfacial mechanisms at the perovskite/HTL or perovskite/ETL interface have been explained by using either HTM or ETM, or sometimes both without an optimized device structure. A few previous attempts employed time-resolved spectroscopy from visible to the microwave range of the spectrum to understand the nature of the excited-states in perovskite solar cells.^20–22^ However, a challenge is still there to understand the evolution of excited states, mobility of photogenerated charges, and the initial charge-transfer to the electron-transport material (ETM), *e.g.*, SnO_2_ or TiO_2_ scaffolds. The same hold for the initial charge transfer to the hole-transport materials (HTM), especially 2,2′,7,7′-tetrakis(*N*,*N*-di-*p*-methoxyphenylamine)-9,9′-spirobifluorene (spiro-OMeTAD).^2^ Here, we aim to understand the specific interfacial processes that follow the absorption of light in the state-of-the-art optimized devices, FTO/SnO_2_/Cs_0.05_(MA_0.17_FA_0.83_)_0.95_Pb(I_0.83_Br_0.17_)_3_/HTL/Au in the near infrared region and on an ultrafast timescale. We introduce a new low-cost diketopyropyrrole‚ (PBDTP-DTDPP-(poly[[2,5-bis(2-hexyldecyl)-2,3,5,6-tetrahydro-3,6-dioxopyrrolo[3,4-*c*]pyrrole-1,4-diyl]-*alt*-[[2,2′-(4,8-bis(4-ethylhexyl-1-phenyl)-benzo[1,2-*b*:4,5-*b*′]dithiophene)bis-thieno[3,2-*b*]thiophen]-5,5′-diyl]])) polymer HTL to improve interfacial electronic interactions with the efficient hole-transfer. Diketopyrrolopyrrole (DPP) pigment has been widely used in optoelectronics,^23,24^ where the whole molecule is almost in one plane because of its fused aromatic conjugated structure, which enhances strong π–π stacking and improves the charge transport properties. In PBDTP-DTDPP, electron donating thiophene and its analogue as a bridge combined with a strong electron deficient DPP core in the conjugated backbone. This results in a strong interaction in the solid state through intermolecular D–A and π–π interactions that is especially beneficial for achieving highly ordered structures at the molecular and microscopic levels. There are few reports on DPP polymer as HTM in perovskite solar cells demonstrating the device characteristics,^25–27^ however no detailed and comparative studies on charge carrier dynamics was performed to date. Our results demonstrate that PBDTP-DTDPP enables 14.73% PCE. The results compared to a spiro-OMeTAD reference device with a 15% PCE show that PBDTP-DTDPP facilitates the ultrafast charge separation in the picosecond regime. However, the nongeminate recombination was slow down when PBDTP-DTDPP polymer was used as HTL in the device suggesting polymeric interlayer for future solar cell applications.

## Experimental

### Materials

Cesium iodide (CsI) (99.999%, Sigma-Aldrich), lead iodide (PbI_2_) (99.999%, Sigma-Aldrich) methyl ammonium bromide (Greatcellsolar), formamidinium iodide (Greatcellsolar), tin chloride dihydrate (SnCl_2_·2H_2_O), (99.99%, Sigma-Aldrich), 2,2′,7,7′-tetrakis-(*N*,*N*-di-4-methoxyphenylamino)-9,9′-spirobifluorene (spiro-OMeTAD) (99% Lumtec), lithium bis(trifluoromethanesulfonyl)imide (Li-TFSI, Sigma-Aldrich), 4-*tert*-butylpyridine (TBP) (96%, Sigma-Aldrich), chlorobenzene (99.9%, spectrophotometric grade) and dimethyl sulfoxide (DMSO) (99.7%, Sigma-Aldrich), *N*,*N*-dimethylformamide (DMF) (99.8%, Sigma-Aldrich), anhydrous ethanol, FTO glass substrates, respectively.

### Device preparation and characterization

To prepare the devices, F-doped tin oxide (FTO) substrates were cleaned with deionized water mixed with surfactant and then with acetone and isopropyl alcohol on an ultrasonic bath for 20 minutes each time. SnO_2_ ETL was spin-coated and annealed at 200 °C for 40 minutes. The (Cs_0.05_(MA_0.17_FA_0.83_)_0.95_Pb(I_0.83_Br_0.17_)_3_) perovskite solution is then prepared in 4 : 1 ratio of DMF : DMSO. The perovskite layer is spin coated over SnO_2_ and annealed at 100 °C temperature for 30 minutes. Then spiro-OMeTAD doped with Li-TFSI and TBP (Sigma Aldrich), was spin-coated over the perovskite layer. The molar ratios for Li-TFSI and TBP were 0.5 and 3.3. In case of PBDTP-DTDPP polymer, undoped sample of 20% w/w is spin coated whereas for doped samples, TBP and Li-TFSI were used. Finally Au electrodes were thermally deposited by vacuum evaporation (<10^−6^ torr). For ultrafast measurements, all the layers have been deposited in similar manner on quartz substrate except the electrodes. Current density–voltage measurement was conducted with a mask in a glovebox under AM1.5 G illumination with an intensity of 100 mW cm^−2^ (Oriel 1 kW solar simulator) using Keithley 4200. SEM image of film was obtained using Hitachi S-4800.

### Cyclic voltammetry analysis

The cyclic voltammetry (CV) data was obtained by using a PowerLab/AD instrument model system with the working electrode (glassy carbon disk), counter electrode (Pt wire), and reference electrode (Ag/Ag^+^) at a 50 mV s^−1^ potential scan speed in a solution of 0.1 M tetrabutylammonium hexafluorophosphate (*n*-Bu_4_NPF_6_)-anhydrous acetonitrile. Film was dropped from a 5.0 mg mL^−1^ warm CB solution onto the glassy carbon working electrode and dried before measurement under the nitrogen stream. With the use of the ferrocene/ferrocenium redox couple (Fc/Fc^+^), the potential of the Ag/AgCl reference electrode was internally calibrated. The HOMO energy level was calculated by using the eqn; HOMO = −(4.80 + *E*_onset_).

### Transient absorption spectroscopy

Transient absorption data were collected using a TAS set-up. TA measurements were performed by using a pump-probe system (UV-VIS HELIOS, Ultrafast systems, Sarasota, FL, USA) and an amplified Ti:sapphire laser. The output of amplified Ti:sapphire laser provides 800 nm fundamental pulses at a 1 kHz repetition rate which were split into optical beams to generate pump and probe pulses. One fundamental beam was used to generate pump beam using an optical parametric amplifier (OPA) system. A white light and near infrared probe was generated by focusing another fundamental beam into a flint glass. Pump and probe beams were focused on a sample and a probe light was collected by a CCD. The instrument response function was ∼100 fs full width at half maximum. The pump wavelength was tuned to 500 nm, produced by an optical parametric amplifier (TOPAS-prime, Light conversion, Lithuania), and the differential change in transmission was detected in the probe range 500 to 1500 nm at several time delays.

### Time-correlated single photon counting (TCSPC)

The TCSPC method was used to record the photoluminescence lifetime profiles of the perovskite incorporated PBDTP-DTDPP polymer and spiro-OMeTAD HTL films. The light source for excitation was the home-built OPO laser. The output of the home-built OPO running in the near infrared region was doubled to generate the excitation pulses at 550 nm. The repetition rate was 500 kHz. A singlet lens was used to focus the excitation pulse onto the sample and the fluorescence was collected with a parabolic mirror. The fluorescence was dispersed with a monochromator, and detected with a single photon detection module. The FWHM of the IRF was 60 ps. Magic angle detection was used to prevent the effects of polarization. All measurements were performed at ambient temperature.

## Results and discussion

**Fig. 1:**
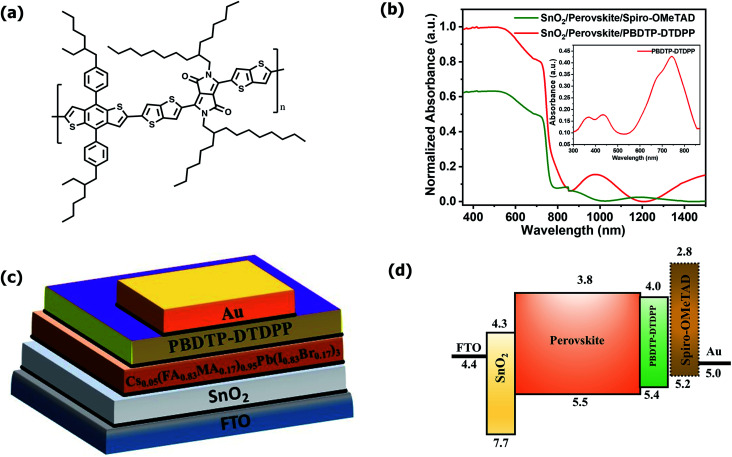
(a). Chemical structure of PBDTP-DTDPP polymer (b) UV-Vis absorption spectra of SnO_2_/perovskite/spiro-OMeTAD, and SnO_2_/perovskite/PBDTP-DTDPP polymer. Inset shows UV-Vis absorption spectra of PBDTP-DTDPP polymer (c) Schematic device structure of perovskite solar cell (d) relative energy levels of different device components in the perovskite solar cell.

The PBDTP-DTDPP polymer was synthesized according to previously reported DPP procedure.^28^ The synthetic steps are described in detail in the ESI Scheme1.†Fig. 1(a) shows the structure of PBDTP-DTDPP polymer molecule. The UV-Vis absorption spectra of PBDTP-DTDPP polymer is given in inset of Fig. 1(b). The absorption spectra of polymer show a pronounced peak at 750 nm and the band of two shoulder peaks at 465 nm and 350 nm. The absorption spectra corresponding to the FTO/SnO_2_/perovskite/HTL films with PBDTP-DTDPP and spiro-OMeTAD are also shown in Fig. 1(b). Fig. 1(c) and (d) show the perovskite solar cell device structure and energy levels of PBDTP-DTDPP material.

The electrochemical properties of the PBDTP-DTDPP polymer molecule are characterized by cyclic voltammetry. From the potential of the first reduction peak, the LUMO was estimated to be −4.04 eV, assuming the absolute energy level of FeCp^2+/0^ to be 4.8 eV below the vacuum level. The HOMO of the PBDTP-DTDPP polymer obtained was −5.43 eV below the vacuum level. The cyclic voltammetry plots are shown in the Fig. ESI1.†

**Fig. 2:**
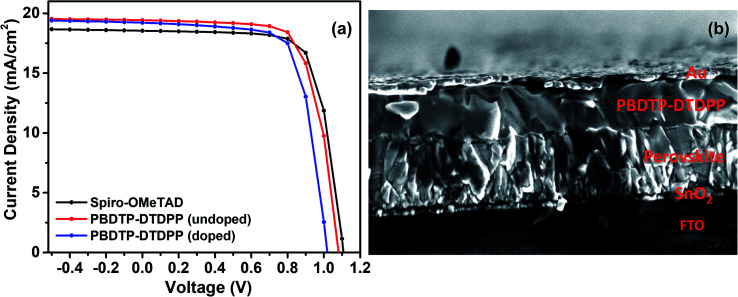
(a) Current–voltage characteristics of spiro-OMeTAD and PBDTP-DTDPP (doped and undoped) HTL incorporated devices. (b) SEM cross section image of perovskite device with PBDTP-DTDPP HTL.

The PSC devices were fabricated using mixed perovskite (Cs_0.05_(MA_0.17_FA_0.83_)_0.95_Pb(I_0.83_Br_0.17_)_3_) as an active layer sandwiched between SnO_2_ ETM and the polymer PBDTP-DTDPP HTL on the top. The current–voltage characteristics are shown in Fig. 2(a). The parameters related to device performances are summarized in Table 1. The achieved PCE for PBDTP-DTDPP (undoped) perovskite device was 14.73% with a short-circuit current (*J*_sc_) of 19.43 mA cm^−2^, an open-circuit voltage (*V*_oc_) of 1.08 V, and a fill factor (FF) of 68.98%. The device results were quite comparable with perovskite/spiro-OMeTAD device that achieved the PCE of 15.02% with *J*_sc_ of 18.54 mA cm^−2^, *V*_oc_ of 1.10 V, and FF of 73.79%. When PBDTP-DTDPP was doped the device PCE was reduced to 14.0% and *V*_oc_ was 1.01 V, and *J*_sc_ was 19.21 mA cm^−2^; and FF of 72.85%. Fig. ESI2† indicates that PBDTP-DTDPP material in perovskite device is still affected by hysteresis although the difference between the forward and reverse current–voltage scan is small. The resulting data is shown in ESI Table 2.† A SEM image in Fig. 2(b) also confirms the adhesion of PBDTP-DTDPP to the perovskite. The SEM images of reference and doped PBDTP-DTDPP in perovskite solar cell devices are shown in Fig. ESI3(a and b).† Notably, the PBDTP-DTDPP polymer enhances the interfacial interaction with the perovskite surface through additional chemical adhesion to improve the interfacial electronic coupling, while keeping the same energetics as that of spiro-OMeTAD.

**Table 1:** Device characteristics of FTO/SnO_2_/perovskite/PBDTP-DTDPP and FTO/SnO_2_/perovskite/spiro-OMeTAD devices^a^

SnO_2_/perovskite/HTL	*V*_oc_	*J*_sc_	FF (%)	PCE
Spiro-OMeTAD	1.10 ± 0.005	18.54 ± 0.30	73.79 ± 0.78	15.02 ± 0.22
PBDTP-DTDPP (undoped)	1.08 ± 0.003	19.43 ± 0.22	68.98 ± 0.96	14.73 ± 0.23
PBDTP-DTDPP (doped)	1.01 ± 0.003	19.21 ± 0.26	72.85 ± 0.94	14.00 ± 0.24

a Average of eight devices (cells) was taken here.

**Fig. 3:**
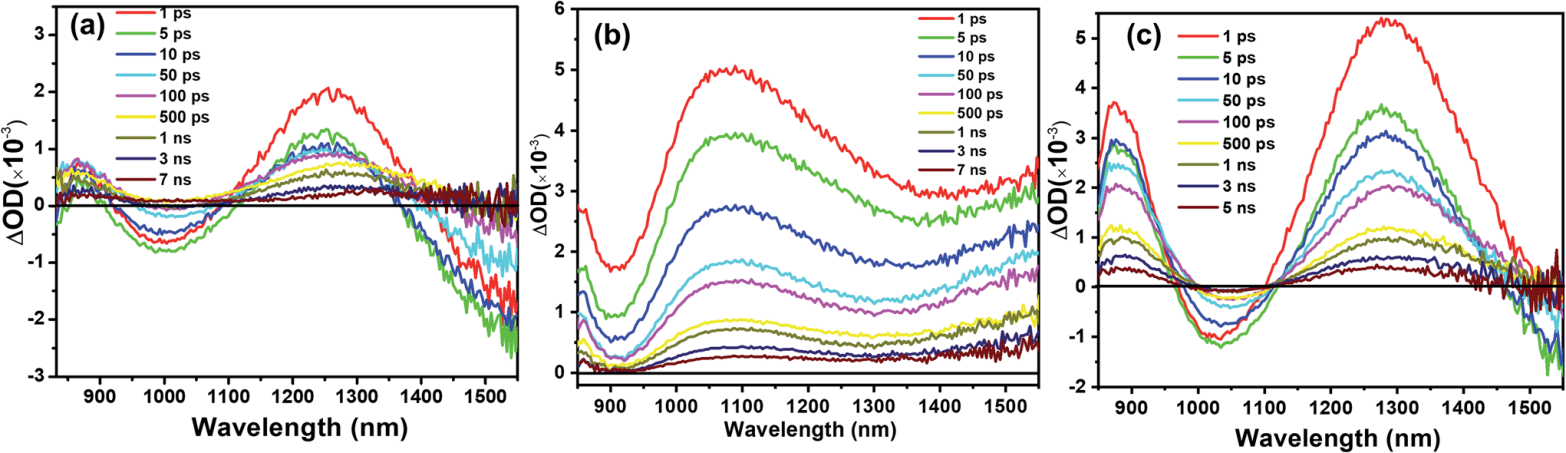
Near-infra red (NIR) excitation state spectrum of (a) SnO_2_/perovskite/(b) SnO_2_/perovskite/spiro-OMeTAD (c) SnO_2_/perovskite/PBDTP-DTDPP polymer. The excitation wavelength was 500 nm and the measured fluence level was 31.5 μJ cm^−2^.

To investigate the charge transport in perovskite devices, transient absorption (TA) spectra of SnO_2_/perovskite/spiro- or PBDTP-DTDPP films were measured using a 500 nm pump with fluence excitation of 31.5 μJ cm^−2^ and the pulse duration was 100 fs. The perovskite domains have been selectively excited at this wavelength and neither spiro-OMeTAD nor PBDTP-DTDPP polymer were excited. The spectra of perovskite with and without HTL are shown in Fig. 3(a–c). The TA measurement of SnO_2_/perovskite/HTL follows the entire light-induced reaction from the generation of charges to the disappearance of the charges. With the aim of understanding the complete SnO_2_/perovskite/HTL carrier mechanism, we probed the dynamics in the near-infra red (NIR) region. This window is yet to be explored in the PSCs. Firstly the photobehavior of the SnO_2_/perovskite was examined and then compared with SnO_2_/perovskite/spiro-OMeTAD and SnO_2_/perovskite/PBDTP-DTDPP cells. Fig. 3(a) shows the excited-state absorption (ESA) ranging from 850 to 1550 nm for SnO_2_/perovskite only whereas, Fig. 3(b) and (c) show the ESA spectra of SnO_2_/perovskite/spiro-OMeTAD and PBDTP-DTDPP polymer, respectively. In Fig. 3(a), a broad excited-state absorption around 1200 nm is observed that disappears at 1430 nm. The absorption band also shows a negative sharp photo-absorption (PA) feature between 950 nm to 1100 nm. This is likely due to band-gap renormalization (BGR) by the photoinduced carriers and trap and defect states as mentioned previously.^29^ In Fig. 3(c), a broad ESA feature is also present. The positive band is red shifted towards 1300 nm and a strong negative band appears between 950 nm to 1300 nm revealing the absorption by the oxidized species of the PBDTP-DTDPP polymer. This also confirms the ultrafast hole transfer at the perovskite/PBDTP-DTDPP interface.^30^Fig. 3(a) and (c) shows a similar feature of negative absorption after ∼1400 nm and in case of spiro-OMeTAD, *i.e.* no negative PA band was noticed in whole of the NIR region which shows its quenched emission due to fast hole injection into spiro-OMeTAD HTL.

The TA kinetics of both spiro-OMeTAD and PBDTP-DTDPP incorporated perovskites have been examined in relation to incident light intensity dependence because the carrier dynamics depends on the charge carrier concentrations. The pump intensity was varied over almost two orders of magnitude from 7.6 to 31.5 μJ cm^−2^, which corresponds to 3.43 × 10^11^ and 2.28 × 10^12^ photons per cm^2^ per pulse, respectively. The decay is found slower at a lower fluence of 7.6 μJ cm^−2^ due to the lower concentration of mobile charges. The TA kinetics were modeled for the two films together and studied according to the reaction scheme given in Table 2.

**Table 2:** The transition and processes, occurring within (a) SnO_2_/perovskite/PBDTP-DTDPP and (b) SnO_2_/perovskite/spiro-OMeTAD films, with their reaction orders. *S*_1_ are excitons generated by light absorption, (e^−1^ + h^+1^) are a loosely bound charge pair

Transition	Rate	Order	Process
*S*_0_ + *hν* → *S*_1_			Photoexcitation
*S*_1_ → [e^−1^:h^+1^]	*k*_1_	First	Charge pair formation
[e^−1^:h^+1^] → e^−1^ + h^+1^	*k*_2_	First	Charge separation
e^−1^ + h^+1^ → S_0_	*γ*_3_	Second	Nongeminate recombination

**Fig. 4:**
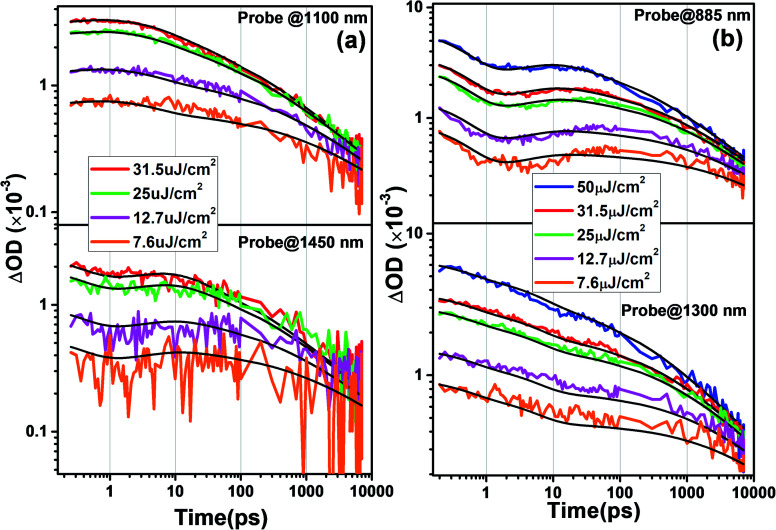
Intensity dependences kinetics of (a) SnO_2_/perovskite/spiro-OMeTAD, and (b) SnO_2_/perovskite/PBDTP-DTDPP polymer. The probing wavelengths were (a) 1100 nm and 1450 nm (b) 885 and 1300 probe wavelengths in successive fluence orders 50, 31.5, 25, 12.7, 7.6 μJ cm^−2^. The pump wavelength was 500 nm. In case of spiro-based films, the fluence 50 μJ cm^−2^ was not taken.

The probe wavelengths for spiro-OMeTAD and PBDTP-DTDPP polymer HTL perovskite were 1100 and 1400 nm, (Fig. 4(a)) and 885 and 1300 nm (Fig. 4(b)). According to the reaction scheme model (Table 1), and the numerical values obtained through reaction steps with rate constants are summarized in Table 3. This fitting model is derived from those used previously for organic solar cells blends.^31,32^ We have fitted the intensity dependences of both spiro-OMeTAD and PBDTP-DTDPP incorporated perovskite films. The model consists of charge formation and charge separation, first order processes with rate constants *k*_1_ and *k*_2_, respectively. Followed at longer time scales by a time-dependent second order recombination rate, *γ*_3_(*t*). The resulting fits are shown in Fig. 4(a) and (b). The charge recombination rates *γ*_3_(*t*) for the non-geminate recombination fits reasonably well for all the three blends with all fluences using the time-dependent second order recombination rate *γ*_3_(*t*) in a model eqn^31,32^ as given below. The actual fitting process allowed for initial concentration dependence of *γ*_3_(*t*) as well, see eqn (1). However, the fitting results with (|S| < 10^−13^) clearly indicates that initial concentration dependence is not present for the samples in this study:1
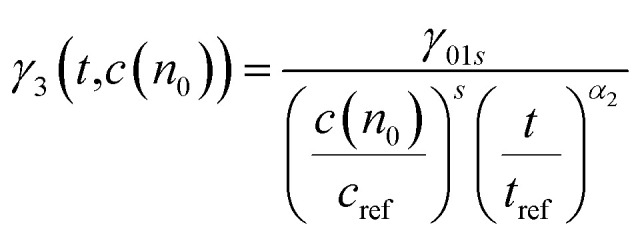
where, *γ*_3_(*t*) is the time-and initial concentration dependent nongeminate recombination rate, *t*_ref_ is 1 ps, *S* is a scaling factor that describes how the initial concentration influences the rate constant (*S* = 0, no influence which is the case in this study, *S* > 0, faster rate for lower fluence, *S* < 0 faster rate for higher fluence), see Table 3, for details, *c*(*n*_0_) is the ΔOD (optical density) at *t* = 0 for each fluence. The results of kinetic modeling for both the HTL incorporated perovskites are compared.

**Table 3:** Modeling results of (a) SnO_2_/perovskite/PBDTP-DTDPP and (b) SnO_2_/perovskite/spiro-OMeTAD films. The rate constants *k*_1_ and *k*_2_ are in s^−1^. Since the extinction coefficients of the species are not known, standard units for the second order rate constants could not be obtained. Instead Abs^−1^ ps^−1^ was used for *γ*_3_. *S* is the intensity dependence of *γ*_3_ whereas, *γ*_3_ is the second order rate constant at *t*_ref_ (1 ps) and *α*_3_ is the time dependence factor for *γ*_3_

Parameters	*k*_1_ × 10^−12^	*k*_2_ × 10^−12^	*γ*_3_ × 10^−12^	*α*_3_
SnO_2_/perovskite/spiro-OMeTAD	2.8	0.21	22	0.55
SnO_2_/perovskite/PBDTP-DTDPP	2.1	0.20	6.8	0.50

The NIR region in Fig. 3(a–c) clearly indicates the charge transfer facilitated further by successive process such as exciton dissociation into mobile charges within the first few picoseconds.^30^ Fig. ESI4(i);† shows an ultrafast electron injection from perovskite conduction band to the SnO_2_ ETL. The positive early peak signal at 850 nm indicates electron absorption on the SnO_2_ and perovskite conduction bands. The excited state spectrum of SnO_2_/perovskite interface and SnO_2_/perovskite/spiro-OMeTAD or PBDTP-DTDPP at 100 fs delay results from generation of charge carriers followed by their cooling. This was also demonstrated previously on fs-time scale.^29,30^ The pump fluence at 500 nm excitation was chosen to be at 7 μJ cm^−2^ in order to avoid multi-exciton interactions. Moreover, Fig. ESI4(ii)† shows the sign of TAS until 5 ps delay which shows negative for FTO/SnO_2_/perovskite whereas positive for SnO_2_/perovskite/spiro-OMeTAD or PBDTP-DTDPP due to electron, hole and oxidized HTL absorption.^30^ Usually oxidation of the HTL is connected with hole migration from the excited perovskite layer to the hole transporter.^29,33^ Therefore dynamics in the NIR region provide additional information of hole transition between two layers. The charge formation and separation rates for spiro-OMeTAD and PBDTP-DTDPP containing films are summarized in Table 3.

The charge formation times are 0.35 ps (*k*_1_ = 2.8 × 10^12^ s^−1^) and 0.47 ps (*k*_1_ = 2.1 × 10^12^ s^−1^), respectively. The charge formation time appears to be faster in spiro-OMeTAD, than in the PBDTP-DTDPP containing films. In an operating solar cell, the bound formed charges must overcome the coulombic attraction and dissociate in order to provide free carrier concentrations of separated charges. The charge separation times for spiro-OMeTAD and PBDTP-DTDPP containing perovskites are 4.8 ps (*k*_2_ = 0.21 × 10^12^ s^−1^) and 5 ps (*k*_2_ = 0.20 × 10^12^ s^−1^), respectively. The charge separation times for both the HTLs are found to be equivalent. The estimated charge separation rates are consistent with previously published reports. It is clear that by changing the fluence levels, both the HTLs obeys intensity dependent kinetics and the rates for charge formation or separation do not vary significantly. To see the recombination in both the HTL containing perovskites, second order kinetic rates were determined that signifies nongeminate recombination. The nongeminate recombination rates (*γ*_3_) for spiro-OMeTAD and PBDTP-DTDPP HTL perovskites are 22 Abs^−1^ ps^−1^ and 6.8 Abs^−1^ ps^−1^, respectively. The relative spectra for the different reaction species are shown in Fig. ESI5(i).† Although, PCE of PBDTP-DTDPP containing device is slightly lower, however the nongeminate recombination rate in PBDTP-DTDPP film appears to be slower than spiro-HTL. This slower rate might have appears due to faster hole injection from the perovskite to the polymer layer.^18,30^ It is believed that the dopants used with the spiro-OMeTAD are deliquescent and hygroscopic, as they degrade the perovskite film as well as the organic HTL, act as recombination centers at the perovskite interface thus diminishing the stability of device. Therefore, our undoped polymeric HTL device has shown almost equivalent device performance compared to spiro-HTL. The results of carrier dynamics demonstrated that PBDTP-DTDPP HTL acts as more effective barrier that prevent the perovskite layer from unusual recombination as well as increased the competence of charge collection by reducing the trap states.

In order to understand the charge transfer and PL quenching of PBDTP-DTDPP layer, time-resolved and steady-state photoluminescence were measured, shown in Fig. ESI5(ii) and ESI6.† The prepared samples, FTO/SnO_2_/perovskite/spiro or PBDTP-DTDPP films were excited at 550 nm by employing time-resolved single photon counting (TCSPC) method. The time-resolved PL given in ESI Table 1† indicates the life times of doped (1.82 ns) and undoped (2.47 ns) which is somewhat lower than that of spiro-HTL (3.07 ns). This verifies that improved charge transfer has occurred in PBDTP-DTDPP polymer compared with spiro-OMeTAD HTL. The PBDTP-DTDPP molecule was also doped similar to spiro-OMeTAD using TBP and LiTFSI. The steady-state PL peak originates at 760 nm as shown in Fig. ESI6† is quenched by the PBDTP-DTDPP device quite well and can have efficient charge transfer compared with the spiro-OMeTAD at same peak wavelength.

## Conclusions

In summary, we demonstrated the effect of polymeric PBDTP-DTDPP HTL on perovskite solar cell through device performance and ultrafast spectroscopy and results were compared with reference spiro-OMeTAD HTL. Although PBDTP-DTDPP HTL obeys nearly similar charge formation or charge separation rates as of reference, however nongeminate recombination time is faster in spiro-than in the polymer HTL. The polymeric PBDTP-DTDPP HTL ends reach to the electrode very well and that can increase its charge collection efficiency thus makes the faster hole-extraction. This investigation is of detrimental importance since it can accelerate the study and implementation of firm dopant-free polymeric hole-transport materials.

## Conflicts of interest

There are no conflicts to declare.

## Supplementary Material

RA-010-C9RA10009A-s001
