# Supplementary material for: Investigating ultrafast carrier dynamics in perovskite solar cells with an extended π-conjugated polymeric diketopyrrolopyrrole layer for hole transportation
Source: RSC Adv. 2020 Feb 12;10(11):6618–24. doi: 10.1039/c9ra10009a (PMC9049750; doi:10.1039/c9ra10009a)
Supplement: RA-010-C9RA10009A-s001 [file RA-010-C9RA10009A-s001.pdf]

Electronic Supplementary Material (ESI) for RSC Advances.
This journal is © The Royal Society of Chemistry 2020

**Electronic Supplementary Information**

# Investigating Ultrafast Carrier Dynamics in Perovskite Solar cells with Extended π-Conjugated Polymeric Diketopyrrolopyrrole Layer for Hole transportation

Chandramouli Kulshreshtha[†*a], Arul Clement[†b],

Torbjörn Pascher[c], Villy Sundström[c], Piotr Matyba*[a]

[a]*Department of Physics, Umeå University, Umeå, 90187, Sweden*

[b]*Swanson School of Engineering, 3700 O'Hara Street, University of Pittsburgh, Pittsburgh, PA 15261, USA*

[c]*Department of Chemical Physics, Lund University, Lund, 22362 , Sweden*

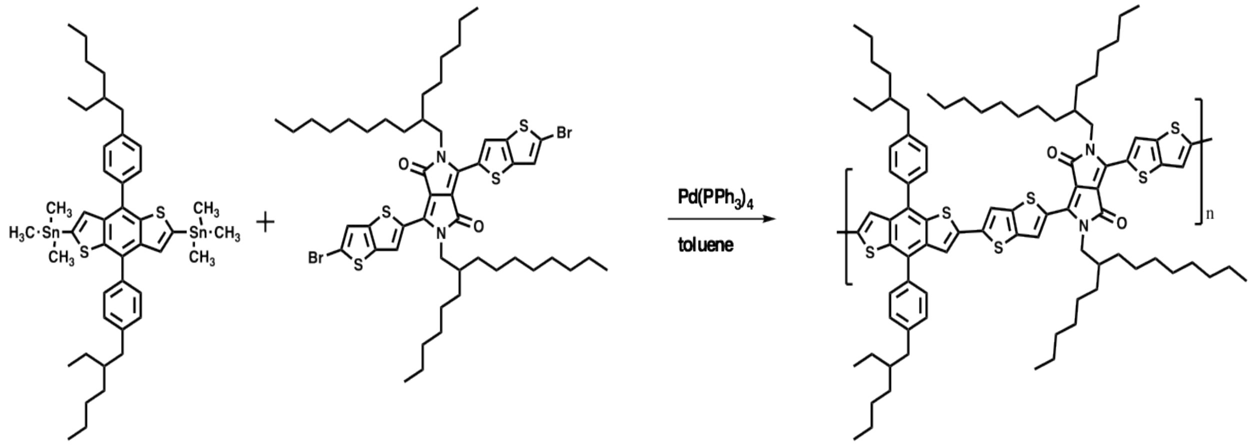


ESI Scheme1: Synthetic route for PBDTP-DTDPP molecule

The synthetic route for PBDTP-DTDPP is shown in Scheme 1. The synthesis of the novel monomers 3,6-bis(2-bromothieno-[3,2-b ]thiophen-5-yl)-2,5-bis(2-hexyldecyl)pyrrolo[3,4-c ]-pyrrole-1,4-(2H ,5H )-dione (DTDPP)[1] and 2,6-Bis(trimethyltin)-4,8-bis(4-ethylhexyl-1-phenyl)-benzo- [1,2-b:4,5-b′]dithiophene (BDT-P)[2] was synthesized according to an previously reported procedure. The polymerization was carried out using $Pd(PPh_3)_4$as a catalyst in anhydrous toluene–DMF to afford a dark green purple solid. The resulting solid was carefully purified through sequential Soxhletextractions using methanol, acetone, hexane and chloroform. The chloroform fraction was concentrated and poured into methanol to afford PBDTP-DTDPP at a yield of 70%.PBDTP-DTDPPexhibits good solubility in chlorinated solvents such as chloroform, chlorobenzene (CB) and 1,2-dichlorobenzene (DCB).The number average molecular weight (Mn) and weight average molecular weight (Mw) of PBDT–CT were found to be 54 kg $mol^{-1}$and 152 kg $mol^{-1}$ respectively, and its polydispersity index (PDI) was found to be 2.81 by using gel permeation chromatography (GPC) with polystyrene standards in chloroform at 40 ºC.

Thermogravimetric analysis (TGA) showed that PBDTP-DTDPPhas excellent thermal stability with a thermal decomposition temperature (Td, at 5% weight loss) of 332 ºC under nitrogen.

Polymerization: The dibromide monomer (DTDPP) (0.5 mmol) and the bis-trimethyltin-monomer (BDT-P) (0.5 mmol) and dry toluene (10 mL): N, N-dimethylformamide (1 mL) were added to a 50 mL two-neck round bottom flask. The reaction container was purged with $N_2$ for 10 min, and then $Pd(PPh_3)_4$ (15 mg) was added. After another flushing with $N_2$ for 10 min, the reactant was heated to 110ºC. The reactant was stirred at 110 ºC for 12 h under the inert atmosphere and then cooled to room temperature. The polymer was precipitated from 100 mL of methanol as small fibrils and then filtered through a Soxhlet thimble, which was then subjected to Soxhlet extraction with methanol, acetone, n-hexane, and chloroform. The polymer was obtained as dark green solid with a yield of 70%. Calculated for $C_{88}H_{114}N_2O_2S_6$: C, 74.31%; H, 8.35%; N, 2.04%; S, 13.98%; found: C, 75.78%; H, 8.65%; N, 1.92%.S, 9.77%.

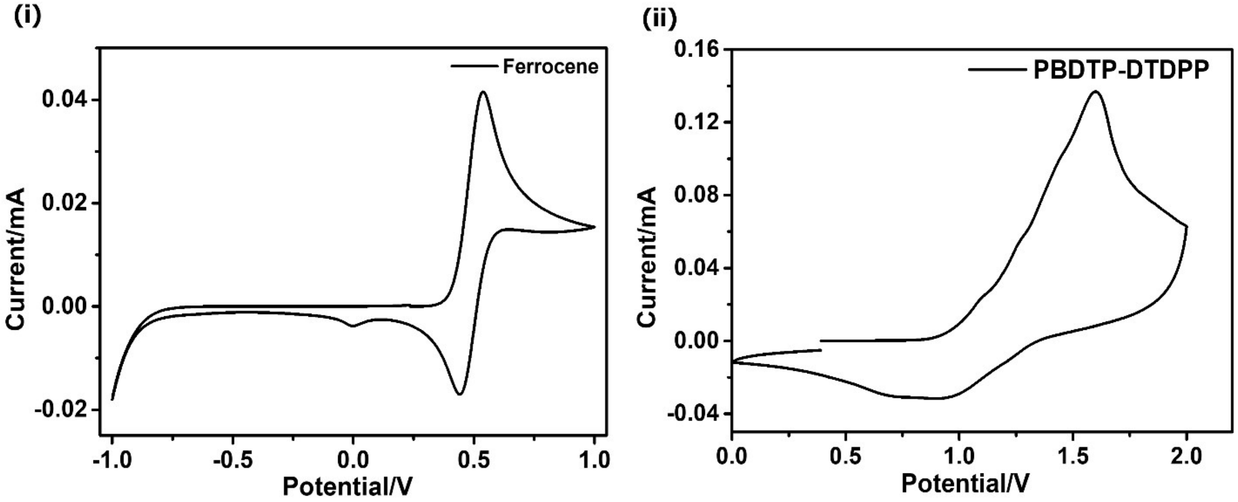


Figure ESI1. Cyclic Voltammetry of (i) Ferrocene standard (ii) PBDTP-DTDPP molecule.

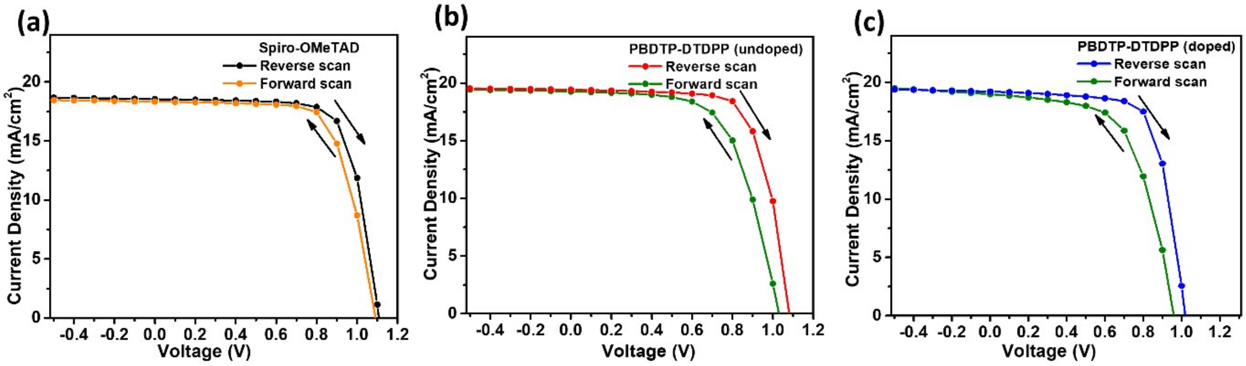


Figure ESI2. Reverse and forward bias scan comparison between (a) FTO/$SnO_2$/perovskite/spiro-OMeTAD and (b), (c) FTO/$SnO_2$/perovskite/PBDTP-DTDPP (undoped and doped) incorporated HTL perovskite devices.

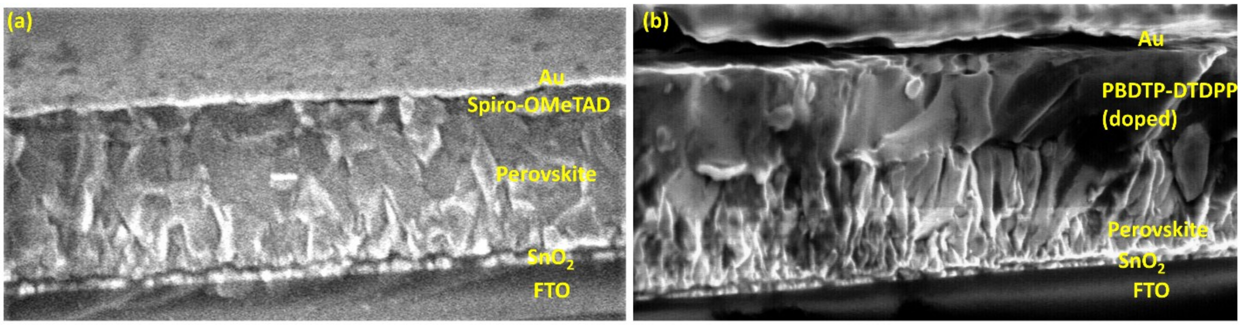


Figure ESI3. SEM images of FTO/$SnO_2$/perovskite/spiro-OMeTAD and FTO/$SnO_2$/perovskite/PBDTP-DTDPP (doped) incorporated HTL perovskite devices.

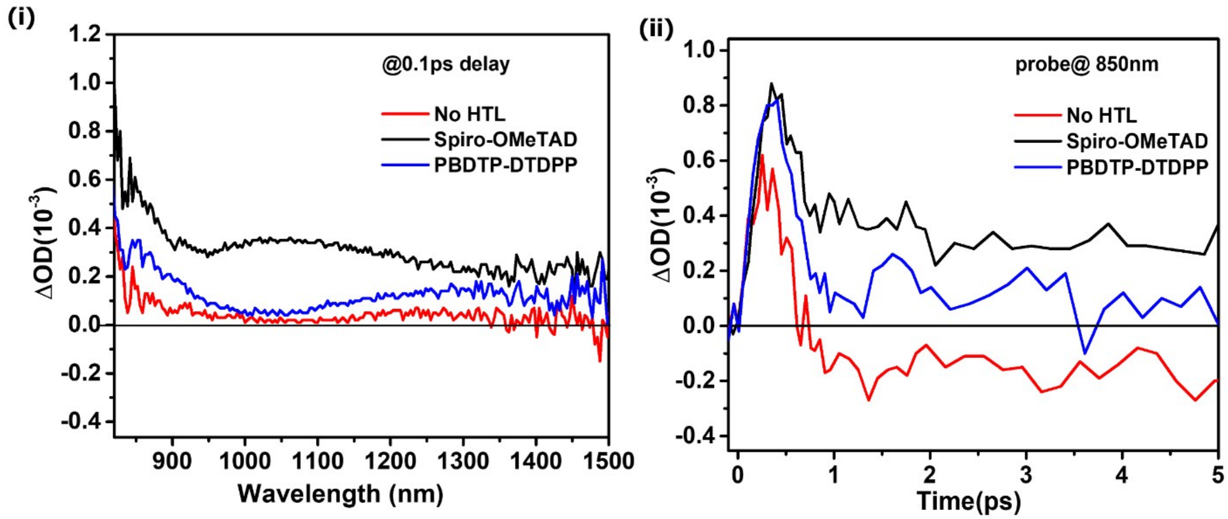


Figure ESI4. (i) Excitation-state spectrum of FTO/$SnO_2$/perovskite, FTO/$SnO_2$/perovskite/Spiro-OmeTAD and $SnO_2$/perovskite/PBDTP-DTDPP at 0.1 ps delay time. The excitation wavelength was 500 nm and the measured fluence level was 7.6μJ/$cm^2$ (ii) Comparative kinetics at 850 nm probe wavelength for FTO/$SnO_2$/perovskite, FTO/$SnO_2$/perovskite/Spiro-OMeTAD and FTO/$SnO_2$/perovskite/PBDTP-DTDPP. The excitation wavelength was 500 nm and the measured fluence level was 7.6μJ/$cm^2$.

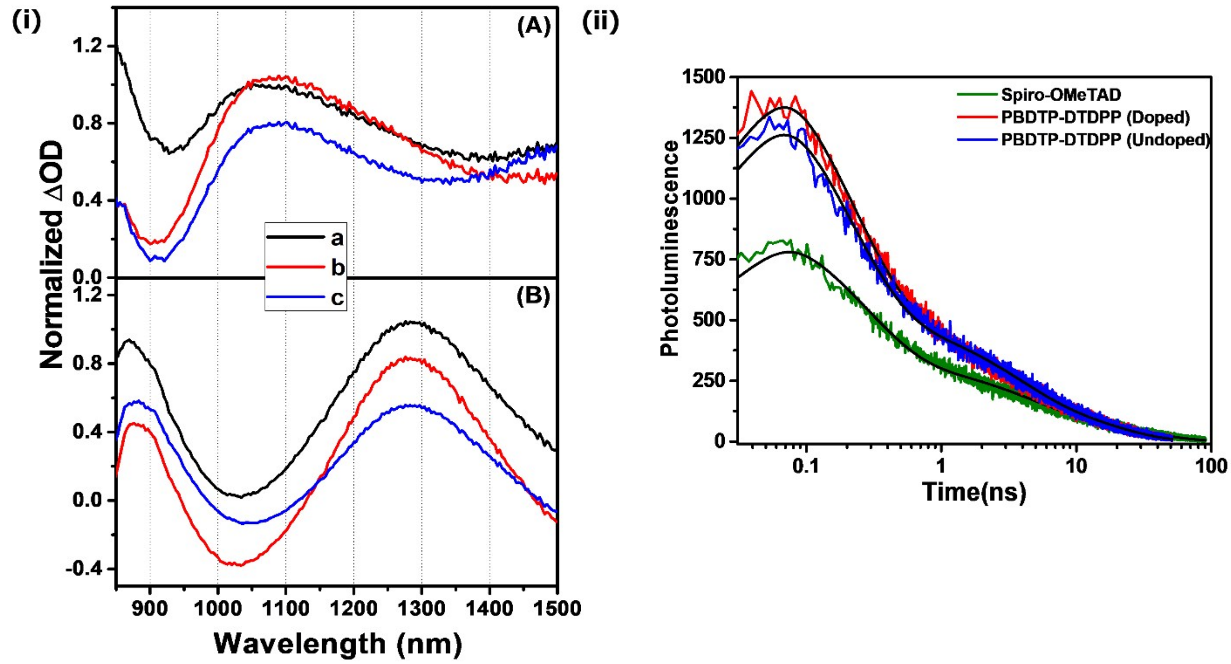


Figure ESI5. (i) Species associated spectra from the fitted model. (A) Spiro-OMeTAD, (B) PBDTP-DTDPP. Species associated spectra shows (a) Spectra of the photo-excited state. (Extinction coefficient normalized to 1 at 1103 nm and 1269 nm for Spiro-OMeTAD and PBDTP-DTDPP, respectively) (b) Spectra of loosely bound charge pair. (c) Spectra of separated charges. (ii) Time-correlated single photon counting (TCSPC) photoluminence of FTO/$SnO_2$/perovskite/spiro-OMeTAD and FTO/$SnO_2$/perovskite/PBDTP-DTDPP (doped and undoped) incorporated HTL films. The excitation wavelength was 550 nm.

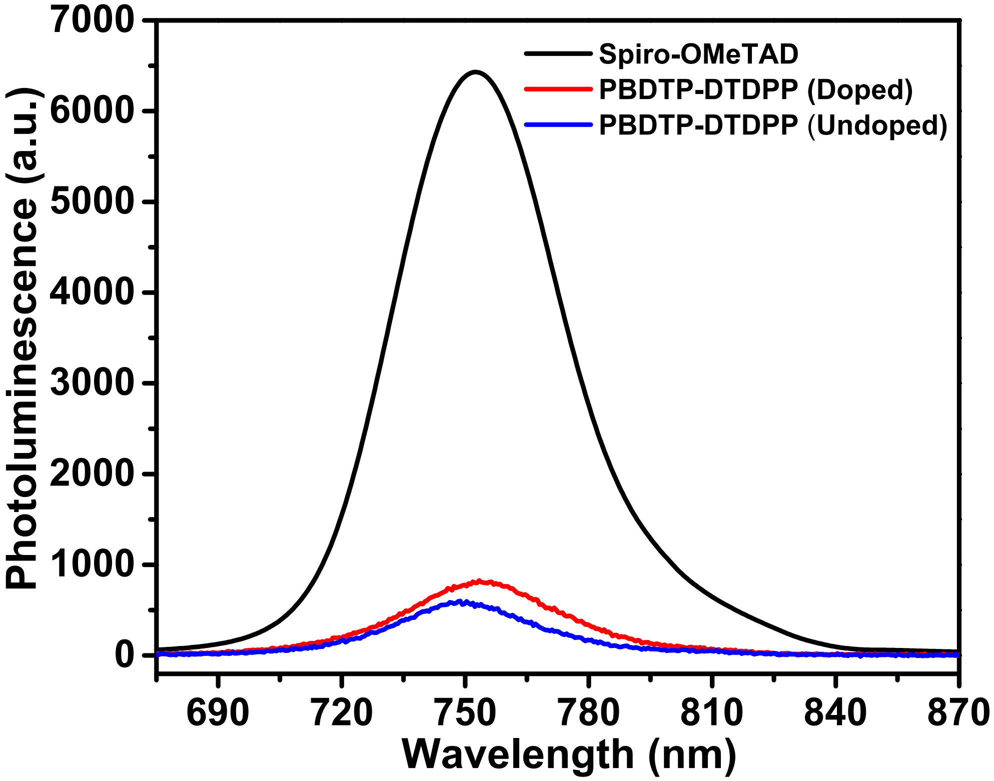


Figure ESI6. Steady-state photoluminence of FTO/$SnO_2$/perovskite/spiro-OMeTAD and FTO/$SnO_2$/perovskite/PBDTP-DTDPP (doped and undoped) incorporated HTL perovskite devices. The excitation wavelength was 550 nm.

**ESI Table 1**. TCSPC fluorescence lifetimes of FTO/$SnO_2$/Perovskite/spiro-OMeTAD and FTO/$SnO_2$/Perovskite/PBDTP-DTDPP (doped and undoped) film. $\tau_1$(fs), $\tau_2$(ps), $\tau_3$(ns) are the fluorescence decay lifetimes whereas $A_1$, $A_2$, $A_3$ are the respective amplitudes of the decay.

| $SnO_2$/Perovskite/HTL | $A_1$ | $\tau_1$ | $A_2$ | $\tau_2$ | $A_3$ | $\tau_3$ |
|---|---|---|---|---|---|---|
| Spiro-OMeTAD | 0.68 | 275 | 0.23 | 3.07 | 0.18 | 19.7 |
| PBDTP-DTDPP (Undoped) | 0.73 | 241 | 0.21 | 1.82 | 0.18 | 11.9 |
| PBDTP-DTDPP (Doped) | 0.76 | 223 | 0.20 | 2.47 | 0.18 | 12.2 |

**ESI Table 2**. Device characteristics with forward and reverse scan comparing hysteresis in spiro-OMeTAD and PBDTP-DTDPP HTL incorporated solar cell devices.

| **Scan** | **HTL** | **Voc** | **Jsc** | **FF (%)** | **PCE** |
|---|---|---|---|---|---|
| Reverse | Spiro-OMeTAD | 1.10 | 18.54 | 73.79 | 15.02 |
| Forward | | 1.10 | 18.35 | 69.91 | 13.95 |
| Reverse | PBDTP-DTDPP (Undoped) | 1.08 | 19.43 | 68.98 | 14.73 |
| Forward | | 1.02 | 19.25 | 63.37 | 12.20 |
| Reverse | PBDTP-DTDPP (Doped) | 1.01 | 19.21 | 72.85 | 14.00 |
| Forward | | 1.00 | 18.98 | 58.47 | 11.10 |

## ESI References


1. Bronstein H.; Chen Z.; Ashraf R. S.; Zhang W.; Du J.; Durrant J.R. Tuladhar P.S.; Song K.; Watkins S.E.; Geerts Y.; Wienk M. M.; Janssen R.A.J.; Anthopoulo T.; Sirringhaus H.; Heeney M.; Mcculloch I. Thieno[3,2-*b*]thiophene−Diketopyrrolopyrrole-Containing Polymers for High-Performance Organic Field-Effect Transistors and Organic Photovoltaic Devices. *J. Am. Chem. Soc.* 2011, 133, 3272–3275.
2. Dou L.; Gao J.; Richard E.; You J.; Chen C.-C.; Cha C. K.; He Y.; Li G.; Yang Y. Systematic Investigation of Benzodithiophene- and Diketopyrrolopyrrole-Based Low-Bandgap Polymers Designed for Single Junction and Tandem Polymer Solar Cells. *J. Am. Chem. Soc.* 2012, 134, 10071−10079.
